# Repair of Tea Polysaccharide Promotes the Endocytosis of Nanocalcium Oxalate Monohydrate by Damaged HK-2 Cells

**DOI:** 10.1155/2020/2198976

**Published:** 2020-04-25

**Authors:** Chuang-Ye Li, Li Liu, Yao-Wang Zhao, Qian-Long Peng, Xin-Yuan Sun, Da Guo, Jian-Ming Ouyang

**Affiliations:** ^1^Department of Urology, Hunan Children's Hospital, Changsha 410007, China; ^2^Institute of Biomineralization and Lithiasis Research, Jinan University, Guangzhou 510632, China

## Abstract

Endocytosis is a protective mechanism of renal epithelial cells to eliminate retained crystals. This research investigated the endocytosis of 100 nm calcium oxalate monohydrate crystals in human kidney proximal tubular epithelial (HK-2) cells before and after repair by four kinds of tea polysaccharides with molecular weights (MWs) of 10.88 (TPS0), 8.16 (TPS1), 4.82 (TPS2), and 2.31 kDa (TPS3), respectively. When HK-2 cells were repaired by TPSs after oxalic acid injury, the cell viability, wound healing ability, mitochondrial membrane potential, percentage of cells with endocytosed crystals, and dissolution rate of the endocytosed crystals increased; the cell morphology recovered; and the reactive oxygen level and lactate dehydrogenase release decreased. Most of the endocytosed crystals were found in the lysosomes. The repair effects of the four TPSs were ranked in the following order: TPS2>TPS1>TPS3>TPS0. TPS2 with moderate MW presented the optimal repair ability and strongest ability to promote endocytosis.

## 1. Introduction

The formation of kidney stones is a complicated process involving crystal nucleation, growth, aggregation, and adhesion [1, 2]. Calcium oxalate monohydrate (COM) is the most usual component of renal stones, and its adhesion to renal epithelial cells is a vital factor in causing stone formation [3, 4]. Crystals adhering to the cell surface are rapidly endocytosed and transferred to lysosomes; the crystals then gradually dissolve in the acidic environment, and the formed calcium and oxalate ions are released outside the cells [5]. Therefore, endocytosis is considered a protective mechanism for renal cells to eliminate retained crystals [6].

Chaiyarit et al. [7] used FITC-labeled COM crystals to observe crystal endocytosis into MDCK renal tubular cells. Fluorescence imaging and flow cytometry results confirmed the endocytosis of the COM into MDCK cells (14.83% ± 0.85%) and the time-dependent reduction of the crystal size. In addition, the endocytosis of a cell depends on its state of health; that is, endocytosis is significantly reduced when cells are damaged [8]. Our previous study [9] showed that the endocytosis of kidney epithelial cells after hydrogen peroxide (H_2_O_2_) damage to nano-COM crystals was weaker than that of normal cells. The endocytosis may increase after repairing the damaged cells.

Plant polysaccharides exhibit numerous biological activities, such as antioxidation, anticancer, antihypoglycemic, and immunomodulation [10–12]. For example, sulfated seaweed polysaccharides can reduce rat kidney damage caused by cyclosporin A (CsA), resulting in decreased lipid peroxidation and increased antioxidant levels [11]. Pillai et al. [12] conducted comet assay (DNA detection) and confirmed that *Ganoderma lucidum* polysaccharides can make the comet parameters of human peripheral blood leukocytes exposed to *γ*-irradiation for 120 min close to the normal levels.

Tea is a favorite natural drink in China. Tea polysaccharides (TPSs) exert antioxidant activity [13]. For example, the protection of Ilex Kuding TPS (IKTP) on vascular endothelial dysfunction and liver injury induced by the high-fructose (HF) level in mice was investigated [14]. The pretreatment of purified green TPS (GTWP) decreased the incidence of H_2_O_2_-induced cell death and inhibited the production of reactive oxygen species (ROS) and malondialdehyde (MDA) induced by H_2_O_2_ treatment [15]. Zhao et al. [16] found that administration of TPS to glyphosate-treated Sertoli cells reduced the levels of MDA and lactate dehydrogenase (LDH), increased the superoxide dismutase (SOD) activity, alleviated cell proliferation inhibition, and inhibited apoptosis induced by glyphosate.

Although the biological activity of TPSs has been widely studied, the relationship between TPSs and kidney stones has been rarely reported. In our previous study [17], four TPSs with MWs of 10.88, 8.16, 4.82, and 2.31 kDa exert antioxidant ability and can repair mitochondria, lysosomes, and intracellular DNA of damaged human renal proximal tubular epithelial (HK-2) cells. TPSs can promote the growth of normal cells and repair damaged cells. TPS2 with moderate MW exhibits the strongest antioxidant ability and repair ability. When HK-2 cells damaged by oxalate were repaired by TPSs, phosphatidylserine (PS) eversion, osteopontin (OPN) expression, and adhesion to the COM crystals decreased [18].

In this research, we further studied the differences in the endocytosis of nano-COM crystals of oxidatively damaged HK-2 cells after repair by four TPSs. Results would provide additional insights regarding the formation mechanism of kidney stones to develop new drugs for treatment.

## 2. Experimental Methods

### 2.1. Polysaccharide Degradation and COM Crystal Preparation

Tea polysaccharide (TPS0) was purchased from Shaanxi Ciyuan Biological Co., Ltd. The average MW is 10.88 kDa. The original polysaccharide TPS0 was degraded using hydrogen peroxide (H_2_O_2_) according to our previous study [17]. The molecular weight (MWs) of degraded TPSs was determined by the Ubbelohde viscosity method at 25 ± 0.2°C. The MWs of TPS1, TPS2, and TPS3 were 8.16, 4.82, and 2.31 kDa, respectively. Calcium oxalate monohydrate (COM) with a size of about 100 nm was prepared according to the previous reference [19].

### 2.2. Cell Culture and Experimental Model

Human kidney proximal tubular epithelial (HK-2) cells were cultured in a DMEM-F12 culture medium (HyClone, Beijing, China) containing 10% fetal bovine serum (HyClone, Beijing, China) and 100 U/mL penicillin-100 *μ*g/mL streptomycin antibiotics (Beyotime, Shanghai, China) at 37°C in a 5% CO_2_ humidified environment. Cell suspension with a concentration of1 × 10^5^cells/mL was inoculated 200 *μ*L, 1 mL, and 2 mL/well in 96-, 12-, and 6-well plates, respectively.

According to our previous research [18], cells were divided into three groups: (1) normal control group: only a serum-free medium was added; (2) damage control group: a serum-free medium containing 2.8 mM oxalate was added and cocultured for 3.5 h; and (3) repair group: a serum-free medium containing 80 *μ*g/mL of TPS0, TPS1, TPS2, and TPS3 was added to repair damaged cells for 10 h.

### 2.3. Hematoxylin and Eosin (HE) Staining

After repair, cells were fixed with 4% paraformaldehyde and then stained with hematoxylin stain (Beyotime, Shanghai, China) for 15 min. After wash, cells were further dyed with eosin (Beyotime, Shanghai, China) for 5 min. The cells in the 12-well plate were observed under an optical microscope (OLYMPUS, CKX41, Japan).

### 2.4. Wound Healing Assay

After repair, a linear trace with a width of about 400 *μ*m was drawn with a sterile 10 *μ*L pipette tip. After washing, a fresh medium was added to continue the culture. The changes of the scratches were observed by an optical microscope (OLYMPUS, CKX41, Japan) at 6 h, 12 h, and 24 h.

### 2.5. Reactive Oxygen Species (ROS) Detection

After repair, cells were stained with 500 *μ*L DCFH-DA (Beyotime, Shanghai, China) for 30 min, the cells in the 12-well plate were observed under a fluorescence microscope (IX51, Olympus, Japan), and the fluorescence intensity of cells in the 96-well plate was detected by a multifunction microplate detector (Synergy H1M, Bio-Tek, USA) at 502 nm.

### 2.6. Lactate Dehydrogenase (LDH) Release Assay

After the repair was completed, each group of the 96-well plate was assayed for OD according to the LDH kit test method. The specific operation is as follows: add 60 *μ*L LDH detection working solution (Beyotime, Shanghai, China) to each well, mix well, and incubate in the dark (about 25°C) for 30 minutes (can be wrapped in aluminum foil and placed in a horizontal shaker or shaken on a rocking bed). Absorbance was then measured by a microplate detector (SafireZ, Tecan, Switzerland) at 490 nm. Dual-wavelength measurements are performed using any wavelength with 600 nm as the reference wavelength.

### 2.7. Measurement of Mitochondrial Membrane Potential (ΔΨm)

After the repair was completed, the samples were stained by JC-1 (Becton, Dickinson and Company, New York, USA) for 15 min. Then, the cells in the 12-well plate were observed by a fluorescence microscope (IX51, Olympus, Japan). The red fluorescence (aggregates) and green fluorescence (monomer) intensities of cells in the 96-well plate were detected by a multifunction microplate detector (Synergy H1M, Bio-Tek, USA) at 585 nm and 514 nm.

### 2.8. Detection of the Percentage of Cells with Endocytosed Crystals

According to our previous study [20], the percentage of cells with endocytosed crystals was detected by a flow cytometer. After reaching the repair time, 200 *μ*g/mL fluorescence-labeled COM dispersed in the serum-free medium was added. After 6 h of incubation, the adherent crystals were removed by complexation with 5 mM EDTA for 5 min. The percentage of fluorescent cells in the 6-well plate was measured by a flow cytometer (FACSAria, BD, USA).

### 2.9. Accumulation of Calcium Oxalate in Lysosomes

After the repair was completed, the culture medium was aspirated, and then, the cells were added with 200 *μ*g/mL fluorescence-labeled COM dispersed in the serum-free medium and incubated for 6 h. The adherent COM crystals were removed by complexation with 5 mM EDTA for 5 min. The lysosomes were stained with 70 nM Lyso-Tracker Red (Beyotime, Shanghai, China) for 2 h. After fixation with 4% paraformaldehyde (Beyotime, Shanghai, China) for 10 min, the cell nucleus was stained with DAPI (Beyotime, Shanghai, China) for 5 min. Crystal accumulation in the lysosome was observed by a laser confocal microscope. The green fluorescence intensity was detected by a microplate detector at 495 nm.

### 2.10. Fluorescence Intensity inside and outside the Cell during Dissolution

The dissolution process of internalized COM crystals was observed and analyzed according to an early report [7]. After reaching the repair time, the cells were added with 200 *μ*g/mL fluorescence-labeled COM and incubated for 6 h. The adherent crystals were removed by the above method. The cells were incubated for 0, 24, 48, and 72 h. The cells were then centrifuged at 1000 rpm for 5 min, and the supernatant (extracellular fraction) was collected. The cells of the 96-well plate were busted by the lysate (intracellular fraction) to measure fluorescence intensity by a multifunction microplate detector (Synergy H1M, Bio-Tek, USA) at 495 nm. A representative TPS2 repair group was selected to observe the intracellular dissolution progress of internalized COM crystals by fluorescence microscopy (IX51, Olympus, Japan).

## 3. Results

### 3.1. TPSs Ameliorated Cell Morphology

**Figure 1:**
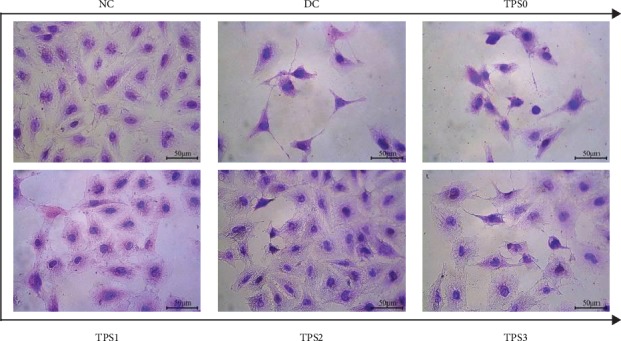
Morphological observation of HK-2 cells before and after TPS repair by an ordinary optical microscope. NC: normal control; DC: damaged control. Oxalate damage concentration: 2.8 mM; damage time: 3.5 h; TPS concentration: 80 *μ*g/mL; repair time: 10 h.

Changes in the cell morphology are related to the cellular damage degree. The cells were stained with HE.  Figure 1 shows the morphological changes in HK-2 cells before and after repair by the four TPSs with MWs of 10.88 (TPS0), 8.16 (TPS1), 4.82 (TPS2), and 2.31 kDa (TPS3).

The nucleus of cells in the normal group is uniform, and the intercellular connections are tight. The number of cells in the injury group decreased, the morphology became disordered, and the connections between the cells were destroyed. After repair by different MW TPSs, the number of cells with full morphology increased; the damaged and condensed cells decreased; and the cells gradually returned to normal.

### 3.2. TPSs Promoted Cell Healing

**Figure 2:**
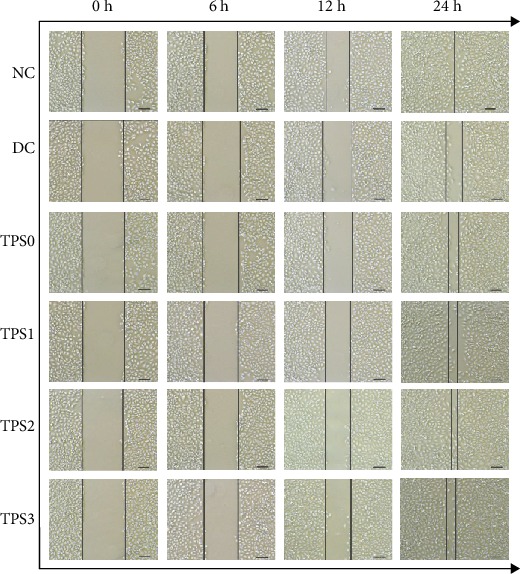
Wound healing abilities of HK-2 cells before and after TPS repair observed under the microscope. Experimental conditions are the same as in Figure 1.

To investigate the healing abilities of damaged HK-2 cells after TPS repair, we conducted a wound healing assay (Figure 2). Cells in the normal group completely healed after 24 h of culture (Figure 2(a)), indicating that the healing ability in the normal group was the fastest. By contrast, the scratches of cells in the injury group healed slowly. The healing abilities of the four repair groups were faster than the injury group but slower than the normal group. The healing ability of TPS2 was the fastest among the repair groups.

### 3.3. TPSs Reduced ROS Levels

**Figure 3:**
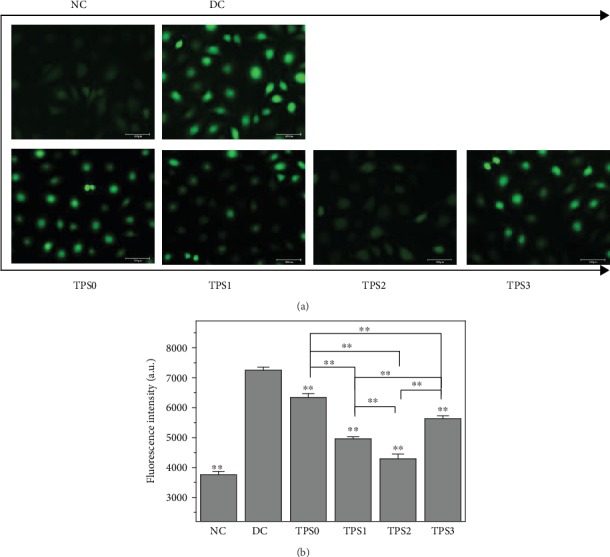
ROS levels in HK-2 cells before and after TPS repair. (a) ROS distribution observed by a fluorescence microscope. (b) Quantitative detection of fluorescence intensity of ROS by a microplate reader. The experimental conditions are the same as in Figure 1. Compared with the DC group, ^∗^*P* < 0.05 indicates a significant difference; ^∗∗^*P* < 0.01 indicates a very significant difference.

Figure 3 shows the ROS levels in HK-2 cells before and after TPS repair. The ROS fluorescence intensity significantly increased after the normal cells were damaged (*P* < 0.01). After TPS repair, the ROS level markedly decreased. The ROS level followed the order of the normal group (3,760)<TPS2 (4,293)<TPS1 (4,957)<TPS3 (5,630)<TPS0 (6,340)<damage group (7,247) (Figure 3(b)). The ROS levels in the cells after TPS2 repair decreased the most, indicating that TPS2 exhibited the strongest repair ability.

### 3.4. TPSs Decreased LDH Release

**Figure 4:**
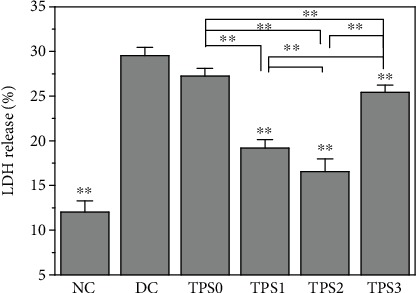
LDH release detection by a microplate reader from HK-2 cells before and after TPS repair. The experimental conditions are the same as in Figure 1. Compared with the DC group, ^∗^*P* < 0.05 indicates a significant difference; ^∗∗^*P* < 0.01 indicates a very significant difference.

Figure 4 shows the LDH release of cells before and after TPS repair. The LDH release of the normal HK-2 cells was 12.02%, and that of the cells damaged by oxalate significantly increased (29.53%, *P* < 0.01). After being repaired by TPSs, the release of LDH decreased to some extent. The percentage of LDH released in the TPS0, TPS1, TPS2, and TPS3 repair groups were 27.24%, 19.18%, 16.55%, and 25.43%, respectively.

### 3.5. TPSs Improved Mitochondrial Membrane Potential (ΔΨm)

**Figure 5:**
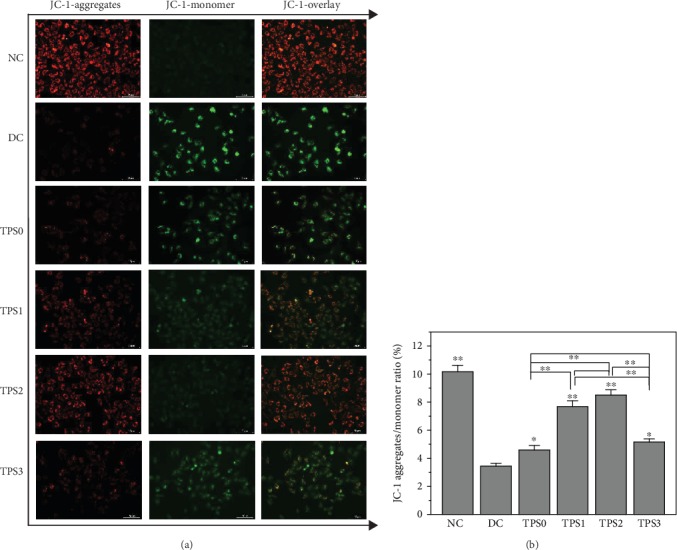
Detection of ΔΨm changes in HK-2 cells before and after TPS repair by a fluorescent probe JC-1 staining. (a) Fluorescence microscopy images. (b) Quantitative fluorescence intensity detected by a microplate reader. The experimental conditions are the same as in Figure 1. Compared with the DC group, ^∗^*P* < 0.05 indicates a significant difference; ^∗∗^*P* < 0.01 indicates a very significant difference.

The change in ΔΨm was determined by observing the transition of JC-1 from red fluorescence to green fluorescence [21].  Figure 5 shows the ΔΨm changes in damaged HK-2 cells before and after TPS repair. Compared with the normal cells, cells in the injury group showed weakened red fluorescence and enhanced green fluorescence, indicating the decrease in ΔΨm. After repair by TPSs, the red fluorescence of the cells was enhanced and the green fluorescence was weakened, indicating the increase in ΔΨm.

### 3.6. TPS Repair Promoted Crystal Endocytosis by Cells

**Figure 6:**
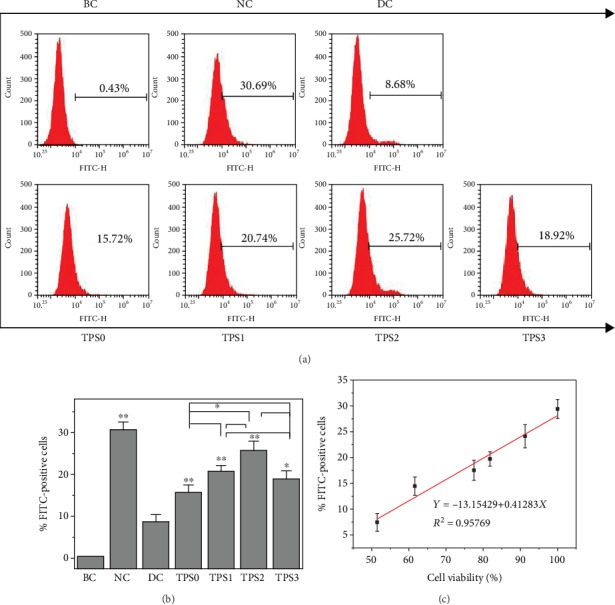
Quantitative detection of the percentage of HK-2 cells endocytosed with nano-COM before and after TPS repair by flow cytometry. (a) Histogram of the percentage of cells endocytosed with nano-COM. (b) Statistical histogram of the percentage of cells endocytosed with nano-COM. (c) The linear relationship of the percentage of cells endocytosed with nano-COM and cell viability. BC: blank control. COM concentration: 200 *μ*g/mL; endocytosis time: 6 h. Other experimental conditions are the same as in Figure 1.

Figure 6 shows the percentage of damaged HK-2 cells with endocytosed crystals before and after TPS repair. The percentage of cells in the normal group that swallowed the crystals reached 30.69%, and that in the injury group only reached 8.68%. The percentage of cells in each TPS repair group ranged from 15.72% to 25.72% (Figures 6(a) and 6(b)). The percentage of cells in the TPS2 group was the highest among the repair groups.

The endocytosis ability of cells is related to their own viability (Table 1). The greater the cell viability is, the stronger their ability to endocytose crystals.  Figure 6(c) shows the relationship between the cell viability and the percentage of cells with endocytosis.

### 3.7. Accumulation of Crystals in Lysosomes

**Figure 7:**
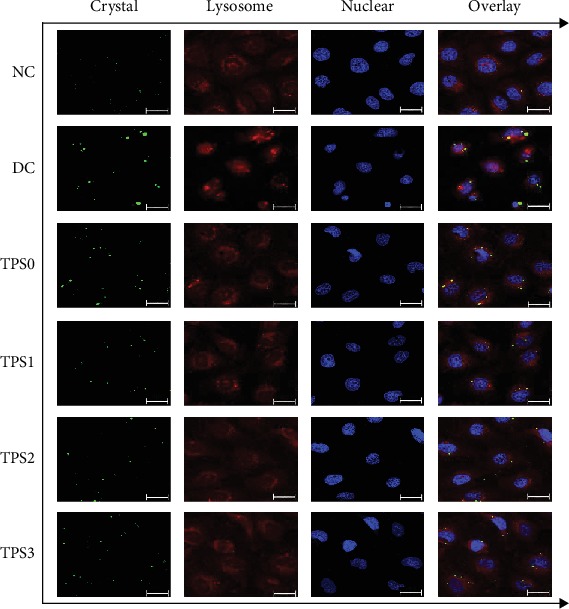
Fluorescence microscope observation of the accumulation of nano-COM in lysosomes of HK-2 cells before and after TPS repair. Green: COM crystals; blue: nuclei; red: lysosomes. COM concentration: 200 *μ*g/mL; endocytosis time: 6 h. Other experimental conditions are the same as in Figure 1. The bars: 20 *μ*m.

Nanocrystals were labeled with FITC-IgG (green fluorescence). DAPI-labeled nuclei (blue), Lyso-Tracker Red-labeled lysosomes (red), and the location of the endocytosed crystals in the cells were observed using a laser scanning confocal microscope. As shown in Figure 7, green fluorescence represents COM crystals and red fluorescence represents overlapping lysosomes, indicating that the endocytic crystals were mainly located in the lysosomes. The nuclei were plump in the normal group, and they shrunk in damaged cells. The nuclei of each repair group are between the normal group and the damaged group, which is similar to the result shown in Figure 1.

In the cells of the NC group, the size of the green fluorescent particles (that is, the nano-COM crystals) is relatively small and the distribution is relatively uniform, indicating that the endocytosed COM crystals have good dispersibility. Due to the limited magnification of the confocal microscope, which is 630 times, many nanocrystals without aggregation or with a small aggregation degree are difficult to observe in the images.

However, in the DC group cells, the number of green fluorescent particles is less than that in the NC group, but the size is larger than that in the NC group. This is possibly because the damaged cell surface expresses more negatively charged adhesion molecules (such as hyaluronic acid, osteopontin, and CD44) [22, 23], and the cell debris generated by damaged cells also contain these adhesion molecules. These cell debris containing adhesion molecules will adhere to the surface of nano-COM with positive surface charges, thus promoting the aggregation of nano-COM crystals [24, 25], leading to a significant increase in the aggregation degree of intracellular crystals endocytosed into the DC group compared with the NC group. Similar results have been reported in previous studies [26, 27].

The endocytosis of cells in each repair group was between the NC group and the DC group.

### 3.8. Dissolution of the Internalized COM Crystals

When FITC-labeled COM crystals entered the lysosomes, the intracellular fluorescence intensity was remarkably enhanced. As the endocytic crystals dissolved into calcium and oxalate ions under acidic conditions in the lysosomes, FITC was released extracellularly [28]. The intracellular fluorescence intensity gradually decreased, whereas the extracellular fluorescence intensity markedly increased. The dissolution of the crystals can be understood by detecting the intracellular and extracellular fluorescence intensities.

**Figure 8:**
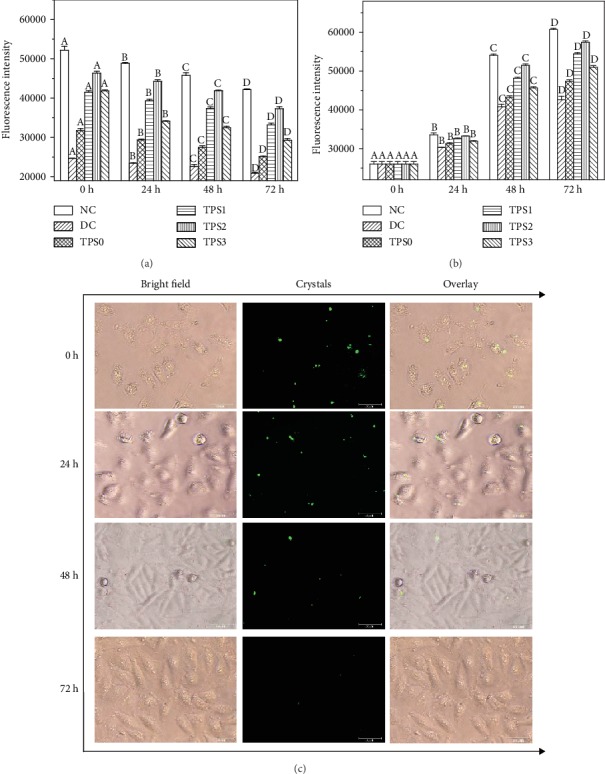
Quantitative detection of the fluorescence intensity of HK-2 cells during crystal dissolution before and after TPS repair by a microplate reader. (a) Intracellular. (b) Extracellular. (c) Representative images of crystal dissolution in the TPS2 repair group observed by a fluorescence microscope. COM concentration: 200 *μ*g/mL; endocytosis time: 6 h. Other experimental conditions are the same as in Figure 1. Different letters (A, B, C, and D) indicate a significant difference (*P* < 0.05) between the normal control (NC), damaged control (DC), and different TPS-treated groups under the same treatment time.

Figures 8(a) and 8(b) show the changes in the fluorescence intensity during the dissolution of the crystals. After 0, 24, 48, and 72 h, the intracellular fluorescence intensity gradually weakened (Figure 8(a)), and the extracellular fluorescence intensity gradually increased (Figure 8(b)), indicating the gradual dissolution of the crystals.

We selected a representative TPS2 repair group to observe the intracellular dissolution process of endocytic COM crystals by fluorescence microscopy (Figure 8(c)). As the time increased from 0 h to 24 h, 48 h, and 72 h, the number of COM crystals showing green fluorescence was gradually reduced, and the crystal size gradually decreased, indicating that the internalized crystals were continuously dissolved.

## 4. Discussion

### 4.1. Repair Mechanism of TPSs

**Figure 9:**
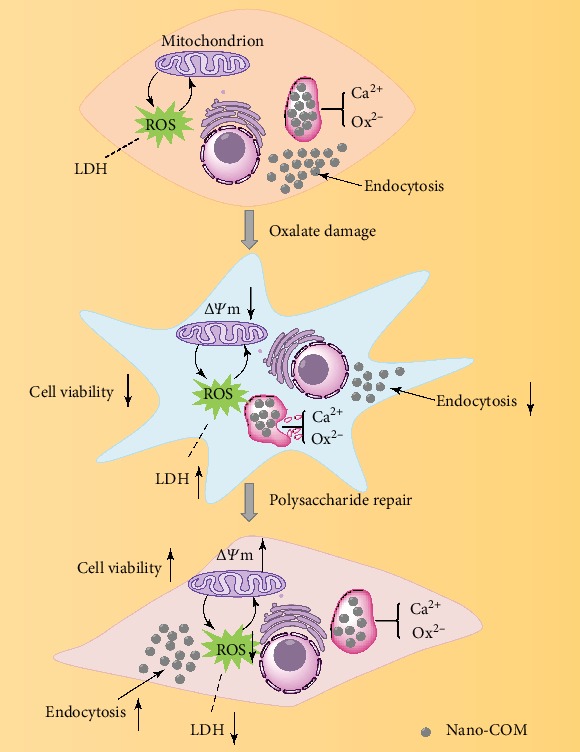
Model of the endocytosis of nano-COM by damaged HK-2 cells before and after TPS repair.

Figure 9 presents a model of endocytosis of nano-COM by damaged HK-2 cells before and after TPS repair. TPSs can increase the viability of damaged cells and the endocytosis ability of the cells to crystals. TPSs can also repair cell morphology, promote cell healing, reduce ROS levels, inhibit LDH release, and accelerate the dissolution of endocytic crystals.

When cells are oxidatively damaged, their ROS levels increase. Excessive ROS beyond the cell's self-clearing ability destroys proteins, carbohydrates, nucleic acids, and lipids in the cell, causing cell damage and even cell death [29]. The ROS produced in the mitochondria interact with unsaturated fatty acids in the mitochondrial membrane to form saturated fatty acids and fatty acid free radicals, resulting in decreased membrane fluidity and ΔΨm (Figure 5) [30]. A significant decrease in ΔΨm causes nuclear condensation (Figures 1 and 7) and secondary generation of ROS [31].

The destruction of the cell membrane causes the release of enzymes in the cytoplasm into the culture medium, thereby increasing LDH release (Figure 4) [32]. TPSs with antioxidant activity can reduce the cell oxidative damage by scavenging harmful free radicals and increasing antioxidant enzyme activity. A large number of studies have shown that the repair of polysaccharides on damaged cells is related to its antioxidant activity [33–35]. For example, Xu et al. [35] found that alloxan caused an obvious decrease in the SOD activity and increase in the MDA level; such effects were inhibited by *Lycium barbarum* polysaccharide (LBP), indicating that it exerted protection against alloxan-induced isolated rat islet ceus damage. Sheng et al. [36] showed that pretreatment of rat pheochromocytoma (PC12) cells with *Platycodon grandiflorum* selenium polysaccharide (PGP1) inhibited the decrease in the cell viability, ROS formation, and apoptotic rate caused by H_2_O_2_. LBPs can also inhibit the H_2_O_2_-induced cell death of endometrial stromal cells (ESCs), decrease the MDA content, and increase the activity of SOD; LBPs also increased Bcl-2 expression and decreased caspase-3 expression [37]. Moreover, LBPs can protect mouse testicular cells against heat-induced injury and H_2_O_2_-induced DNA injury by improving the SOD activity [38].

### 4.2. Mechanism of TPSs in Promoting the Endocytosis of Nano-COM Crystals

Endocytosis is the process by which cells absorb various substances from the external environment [39] and can be divided into pinocytosis and phagocytosis [40]. Phagocytosis is a special endocytic pathway that occurs primarily in phagocytic cells, such as monocytes, neutrophils, and macrophages [41]; pinocytosis occurs in almost all eukaryotic cells [42, 43].

The endocytic pathway of renal cells to calcium oxalate crystals is affected by cell type and crystal properties. Micron crystals are endocytosed by macropinocytosis, whereas nanocrystals enter cells through classical clathrin-mediated endocytosis [44]. For example, COM crystals with a size of 3–5 *μ*m are mainly internalized into MDCK cells by macropinocytosis [45]. In our previous study [46], we have observed that 50 and 100 nm sized COM and COD were mainly internalized via the clathrin-mediated pathway, whereas 1 *μ*m COM and COD were mainly internalized by macropinocytosis.

Endocytic crystals are rapidly transferred to the lysosomes to dissolve into oxalate and calcium ions (Figures 7 and 8); hence, endocytosis is a defense mechanism of cells to adhering crystals [6]. However, the endocytosis ability of cells to crystals is limited. The amount of endocytic crystals exceeds the range that the cells can dissolve, leading to lysosomal rupture, nuclear shrinkage, and mitochondrial membrane potential decrease.

### 4.3. Effect of Molecular Weight

Among the four TPSs evaluated, TPS2 with moderate MW exhibited the greatest promoting effect on endocytosis of the crystals in injured cells. Polysaccharides with high MW (such as TPS0) are bulky and cannot enter cells across the membrane [47, 48]. For example, Qi et al. [49] found different MW sulfated polysaccharides extracted from Ulva pertusa Kjellm have antioxidant activity, but polysaccharides with higher MW showed lower activity. Similarly, the antioxidant abilities of chlorophyll polysaccharides with MWs of 2,918, 256.2, 60.66, and 6.55 kDa were compared; the results showed that the polysaccharide with the largest MW had the lowest activity [50].

When the polysaccharide's MW is too small (such as TPS3), the sugar chain is short and the hydrogen bonding is weak; as such, an active helical structure cannot easily form. Thus, excessive ROS in the damaged cells cannot be effectively removed, the repair effect is poor [51], and the ability to promote endocytosis is weak. Liao et al. [52] also showed that polysaccharides with moderate MW of 1 × 10^5^ − 4 × 10^5^ Da have the highest activity, while those with low MW (5 × 10^3^ − 1 × 10^4^ Da) have no biological activity. Sasaki et al. [53] found that a mushroom polysaccharide with MW of 1.6 × 10^5^ kDa exerts an antitumor activity, and that with MW less than 5 × 10^3^ kDa has no antitumor activity.

In general, polysaccharides with moderate MW have the highest activity. For example, Yuan et al. [54] demonstrated that three polysaccharides from *Ligusticum chuanxiong* Hort with MWs of 12.3, 28.3, and 63.1 kDa exert antioxidant activity; the polysaccharide with moderate MW exhibits the strongest antioxidant activity. You et al. [55] studied the protective effects of *Lentinus edodes* polysaccharides with MWs of 25.5, 306.2, and 605.4 kDa on D-galactose-induced oxidative stress-induced myocardial cells in mice; the polysaccharides with moderate MW exhibited the strongest protective effects.

Plant polysaccharides with different MWs exhibit biological activity of different degrees due to differences in monosaccharide composition and acid group type and content. In the present study, the difference in the repair effect of TPSs is mainly affected by MW given their similar main chain structure and carboxyl content.

## 5. Conclusion

The four TPSs evaluated can improve cell viability, repair cell morphology, accelerate cell wound healing, reduce the ROS level, inhibit LDH release, increase the mitochondrial membrane potential and dissolution rate of endocytic crystals, and promote endocytosis. TPS2 with moderate MW exhibits the optimal performance in promoting the endocytosis of nano-COM crystals by HK-2 cells. Hence, TPSs may be potential drugs to prevent the formation of kidney stones.

## Figures and Tables

**Table 1:** Cell viability of damaged HK-2 cells before and after TPS repair.

	NC	DC	TPS0	TPS1	TPS2	TPS3
Cell viability (%)	100 ± 1.7	51.47 ± 0.9	61.59 ± 1.3	81.83 ± 2.7	91.26 ± 1.8	77.5 ± 2.2

NC: normal control; DC: damaged control. Oxalate damage concentration: 2.8 mM; damage time: 3.5 h; polysaccharide concentration: 80 *μ*g/mL; repair time: 10 h.

## Data Availability

The data used to support the findings of this study are available from the corresponding author upon request.
